# Keep Healthcare Workers Safe: Application of Teleoperated Robot in Isolation Ward for COVID-19 Prevention and Control

**DOI:** 10.1186/s10033-020-00464-0

**Published:** 2020-06-09

**Authors:** Geng Yang, Honghao Lyu, Zhiyu Zhang, Liu Yang, Jia Deng, Siqi You, Juan Du, Huayong Yang

**Affiliations:** 1 State Key Laboratory of Fluid Power and Mechatronic Systems, School of Mechanical Engineering, Zhejiang University, Hangzhou, 310027 China; 2 Department of Gastroenterology, First Affiliated Hospital of Zhejiang University School of Medicine, Hangzhou, 310003 China

At the time of writing, the coronavirus disease (COVID-19) has affected 212 countries and territories across the globe. According to the world health organization (WHO), a total number of 4,735,622 confirmed cases, including 316,289 deaths was reported [1]. In the fight against COVID-19, nurses, doctors, and other healthcare workers are in the front line of the battle bearing the higher risk of infection [2]. The International Council of Nurses (ICN) gathered further information to suggest that more than 90,000 healthcare workers have been infected worldwide [3]. Personal protective equipment (PPE) shortage is one of the key factors increasing the infection risk for the medical staffs. Therefore, finding alternative ways to lower the infection risk has become an urgent problem to be solved.

Using robotic technology [4] and telemedicine [5] to help with the combat of COVID-19 outbreak has gained a great attention for good reasons: more robots and virtual meetings in the field means less person-to-person contact, thus, lower risk of infection for healthcare workers. Using robots can also reduce community transmission and PPE consumption.

To win the battle, health and wellness of every healthcare worker has to be guaranteed [6]. From a requirements elicitation survey that was conducted at the early stage of the pandemic by interviewing the healthcare workers in the First Affiliated Hospital of Zhejiang University School of Medicine (FAHZU, the designated hospital for diagnosis and treatment of COVID-19 in Zhejiang Province, China), the following routines in the isolation ward are especially time-consuming or difficult while wearing PPE and are welcomed to be replaced using robotic devices: (1) Daily checkups on patient’s physical and mental conditions, (2) Delivery of medicine, food, or other essential items, (3) Operation of the medical instruments, (4) Extensive disinfection of the high-touch surfaces, (5) Auscultation while wearing PPE.

Moreover, even with PPE, healthcare workers can still be infected in some special cases [7]. Therefore, a teleoperated device that can perform the basic routines in the isolation ward can not only reduce the risk of infection but also ensure healthcare workers have enough time for more important tasks.

Based on the needs mentioned above, a telerobotic system for remote care operation in isolation ward is developed and introduced here. The research and development of this system are mainly focused on the robotic design, motion capture, mapping algorithm development, telepresence software development, and control strategy design.

**Figure 1:**
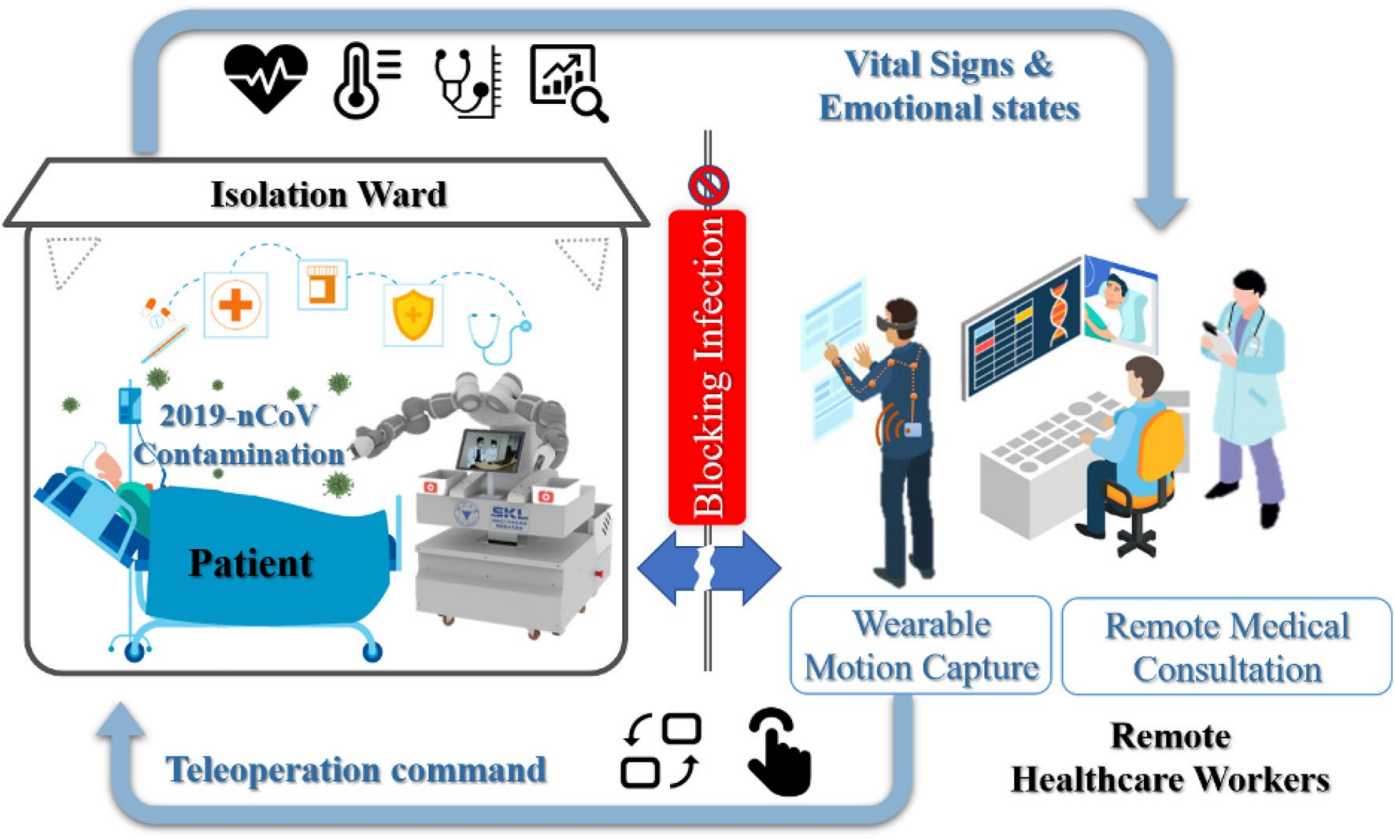
Schematic of the developed telerobotic system (Vectors of the patient and caregivers were designed by Freepik)

The proposed telerobotic system (Figure 1) has two main subsystems: the teleoperation system and the telepresence system:

1. The **teleoperation system** consists of a wearable initial motion capture device and a dual-arm collaborative robot (YuMi, IRB14000). Using the motion capture device, upper limb motion data of the healthcare worker can be obtained and used to control the robot arm motion remotely. A pair of data gloves are used to capture the finger motions and teleoperate the grippers or other end effectors of YuMi.
2. The **telepresence system** is achieved by a tablet computer attached to the front of the teleoperated robot. A Multi-Users Audio/Video Conference System for remote medical consultation is developed and deployed based on Web Real-Time Communication (WebRTC). A voice wake-up function is developed to facilitate patient’s operation, and it reduces the chance of contact between patient and the robot. Furthermore, a deep neuron network is used to monitor the patient’s emotional states by deploying on the tablet computer.

The proposed system aims to block infections by reducing the chance of contact between the patients and the healthcare workers. With the two subsystems, the teleoperated robot can assist or even replace the medical staffs to take care of patients in the isolation ward. In other words, the teleoperated robot becomes the healthcare worker’s eyes, ears, and body in the isolation ward.

**Figure 2:**
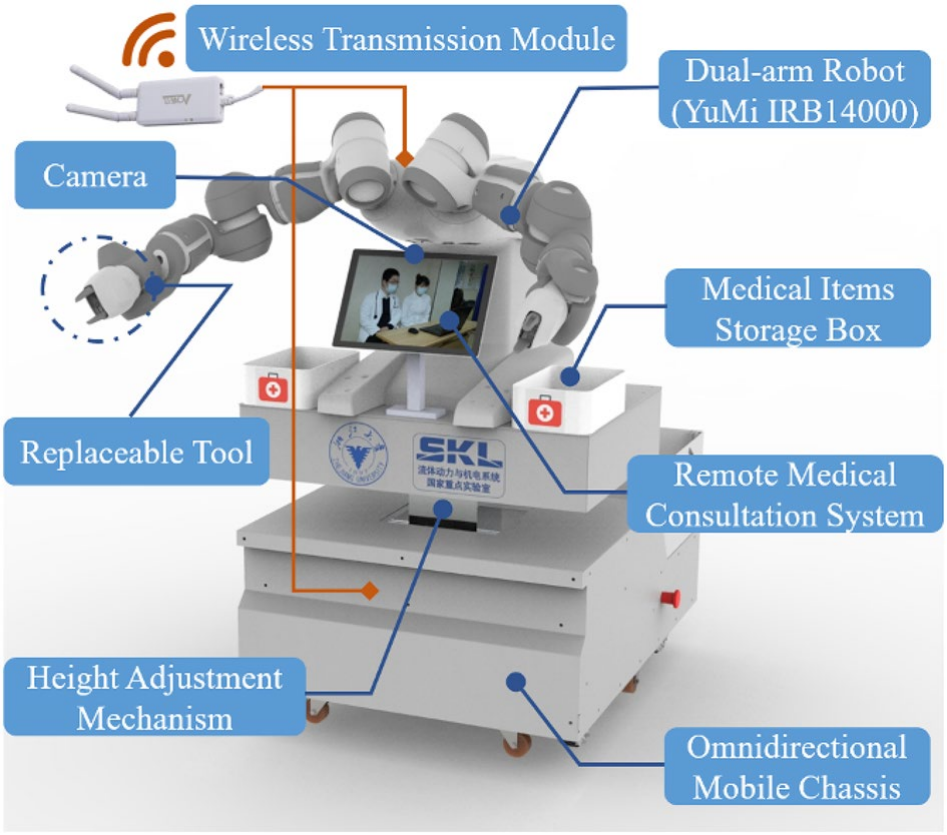
Structure of the self-designed teleoperated robot

There are four main parts of the self-designed teleoperated robot (Figure 2): omnidirectional mobile chassis, dual-arm collaborative robot above the chassis, height adjustment mechanism, and the other support devices. The chassis can move in all directions through different motion combinations of four Mecanum wheels, which is suitable for flexible movements in the narrow space of the isolation ward. The collaborative robot, Yumi, produced by ABB is chosen as the manipulator for teleoperation. An electric height adjustment mechanism is designed between the dual-arm manipulator and the mobile chassis, so that the height of YuMi can be adjusted to ensure the workspace for unstructured operation cases. In order to meet the confirmed needs of patient care, special replaceable connectors for various end effectors (Stylus Pen, Doppler ultrasound equipment, Handheld disinfection equipment, etc.) are designed. A storage box for medicine, disinfectants and other equipment is installed on the side of the robot. A tablet computer is installed in the front of the robot, which is used to conduct the remote daily medical checkups. Patients’ emotional state monitoring is also achieved based on the face data acquired using the camera on the tablet PC. Remote control is achieved via a pair of mini WiFi repeaters between the robot and the healthcare worker.

For robot control, a wearable motion capture suit consists of 18 inertial measurement units (IMUs) is chosen to capture the healthcare worker’s motion data. Teleoperation is achieved using incremental motion mapping between the operator and robot, which is intuitive and convenient. It allows healthcare workers to reproduce their professional skills through the robot more effectively with lesser training needed. A pair of data gloves are used to collect the bend angle of the healthcare worker’s finger during the teleoperation. Various gesture instruction rules are designed to control the end effectors.

This research is approved by the Clinical Research Ethics Committee of FAHZU. Currently, the proposed telerobotic system is undergoing clinical trials. Functional verification has been completed at the Teaching and Research Center of FAHZU and the proposed teleoperated robot has been tested in the emergency center’s Intensive Care Unit (ICU) of FAHZU.

**Figure 3:**
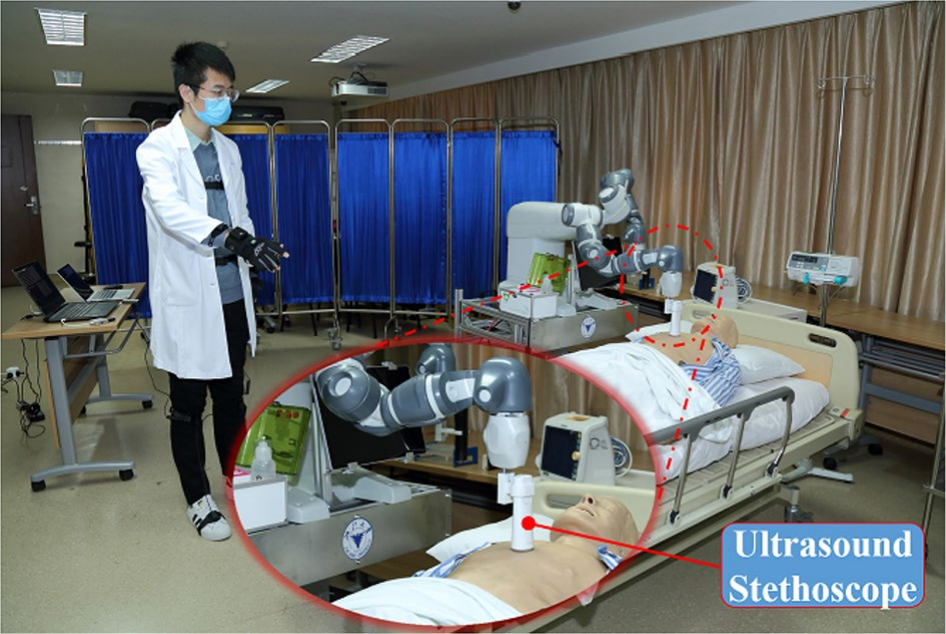
Remote auscultation using a Doppler Ultrasound Stethoscope based on the teleoperated robot

**Figure 4:**
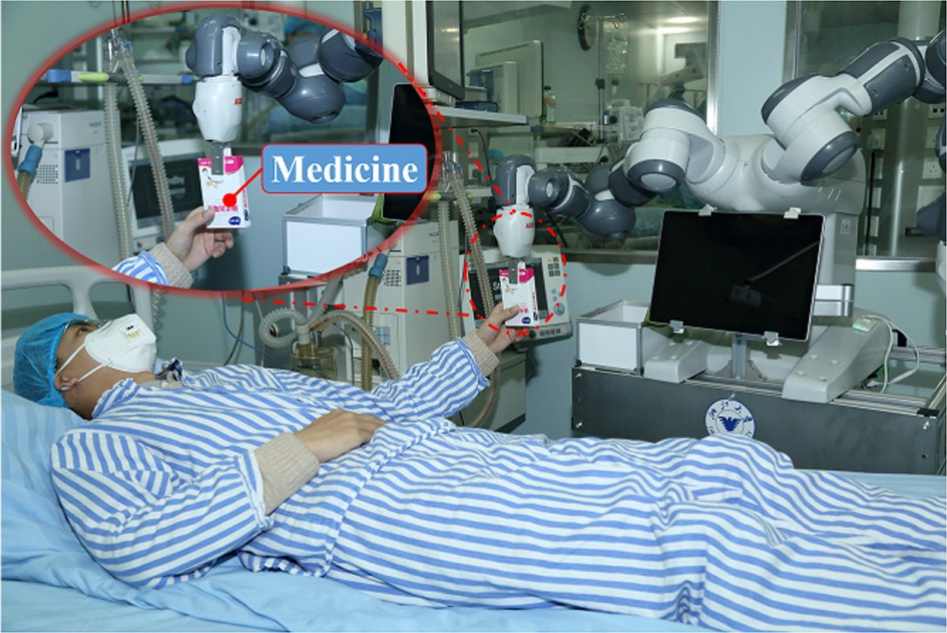
Remote medicine delivery using the teleoperated robot

**Figure 5:**
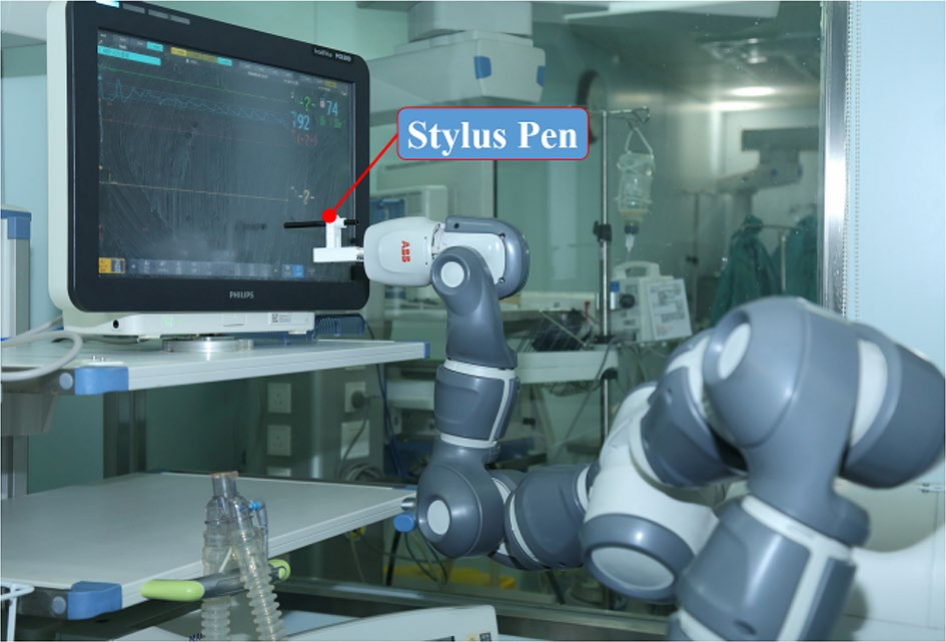
Remote operation of the medical instruments using the teleoperated robot

**Figure 6:**
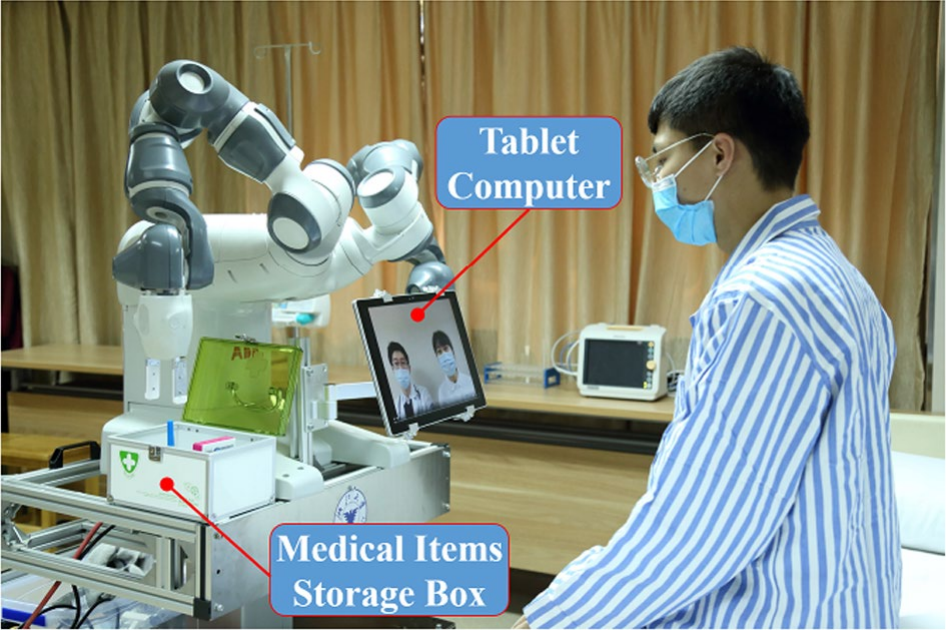
Remote daily consultation of physical and mental conditions using the telepresence system

**Figure 7:**
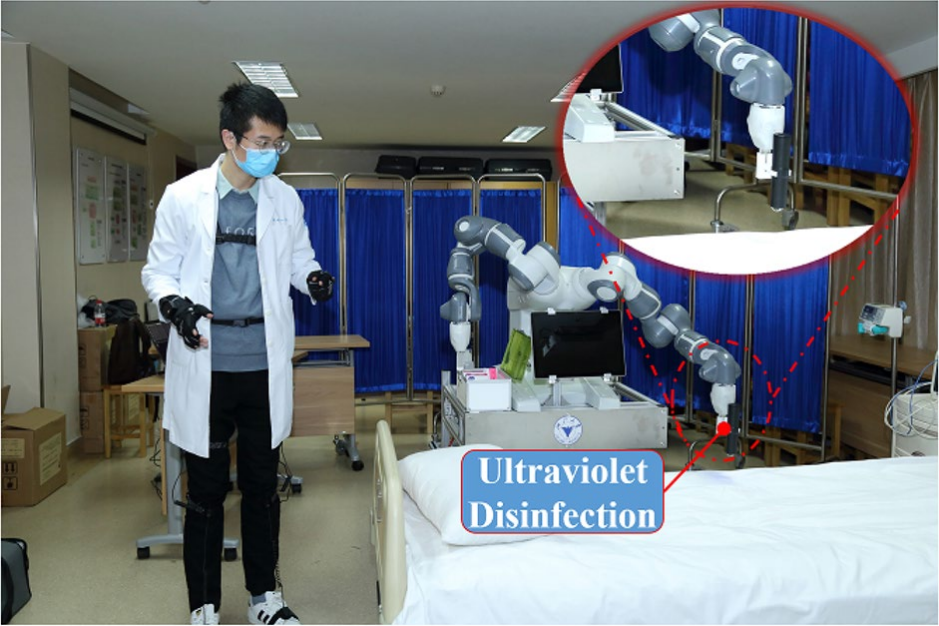
Remote extensive disinfection of the high-touch surfaces using the teleoperated robot

Remote auscultation is achieved using a Doppler Ultrasound Stethoscope attached to the end of the robot arm, which enables the healthcare workers to auscultation outside of the isolation ward (Figure 3). To achieve the remote object delivery, the two-finger gripper of YuMi is used to grip the medicine or other medical necessities from the medical storage box and then delivers it to the patient (Figure 4). A custom-designed Stylus Pen attached to the end of the gripper’s finger is used to operate the medical instruments remotely (Figure 5). The remote daily checkups are conducted via the tablet PC in the front of the robot, which allows the healthcare workers to communicate with the patients with no physical contact (Figure 6). Last but not the least, remote extensive disinfection of the high-touch surfaces is achieved by a custom-designed handheld ultraviolet disinfection device attached to the end of the gripper (Figure 7).

To summarize, this newly designed telerobotic system combines the strengths of healthcare workers (expert knowledge for patient care) with the strengths of robotics (social distancing and capabilities to work in hazardous environments) to give the best outcome to patients and healthcare workers. Implementation of the robot in the battle against COVID-19 has obtained positive feedback from healthcare workers for its potential in blocking infection and effectiveness in relieving medical workers from repeated tasks. In addition, this teleoperated robot also has potentials in other areas like space exploration, bomb disposal, disaster rescue and any other fields with a need of remote operation.
